# Therapist experiences working with Asian American college students

**DOI:** 10.1080/28324765.2024.2338052

**Published:** 2024-04-09

**Authors:** Lisa Liu, Natasha Thapar-Olmos, Joey Fung, Lorinda Ho, Anna Lau

**Affiliations:** Department of Psychology, University of California, Los Angeles, CA, USA

**Keywords:** Asian American, cultural competence, qualitative, treatment, practice-based evidence

## Abstract

This qualitative exploratory study examined therapist experiences in working with Asian American clients at a university counseling center. Individual semi-structured interviews were conducted with 17 licensed therapists of diverse ethnic backgrounds to explore the presenting concerns and treatment approaches used in their work with Asian American clients. Data were analyzed within a consensual qualitative research (CQR) framework to identify themes informed by the research questions. Family stress was the most frequently reported presenting problem, followed by identity search, emotion regulation, academic stress, stereotypes and discrimination, and acculturative stress. Therapists discussed insight-oriented and directive approaches with equal frequency and often integrated approaches according to the needs of the client. Most therapists reported discussing ethnic match in therapy, and perceptions of ethnic match as favorable or unfavorable seemed to vary across cases. Findings highlight the value of research in delivering culturally responsive treatments for Asian Americans.

Asian American college students appear to be at particular risk for developing mental health problems. Research has found that Asian American college students report higher levels of depression and anxiety (Kalibatseva et al., 2017; Lau et al., 2009), overall distress (Locke et al., 2012; Stokes et al., 2021) and suicidal ideation (Kisch et al., 2005) compared to their European American peers. Despite greater mental health needs, Asian American college students tend to underutilize mental health services (Eisenberg et al., 2007; Kearney et al., 2005) due to stigma towards mental illness and other cultural barriers (Gee et al., 2020; Miller et al., 2011). The “model minority” stereotype of Asian American students attaining high achievement may also cause others to overlook or underestimate their mental health needs (Guo et al., 2014; Sue et al., 2012).

To address these risks, it is important to understand the unique cultural, familial, and contextual stressors that Asian American college students face. These may include academics, family conflict, family obligations, acculturative stress, and the challenges of navigating biculturalism (Fuligni, 2007; Liu et al., 2012; Nguyen et al., 2018). For instance, Asian American college students have reported more frequent racial discrimination experiences compared to White students (Huynh & Fuligni, 2012; Pieterse et al., 2010), and others have found that racial discrimination experiences contributed to depression, anxiety, somatic and trauma symptoms among Asian American college students (Chen et al., 2014; Hwang & Goto, 2008; Pieterse et al., 2010; Tummala-Narra et al., 2018). Asian immigrant college students may also struggle with acculturative stress, including language barriers and the stress of acculturating to a new environment and the dominant culture (Han et al., 2017; Wong et al., 2017). While the perception and value of family closeness can have positive effects on academics, Asian American students reported struggling to balance school with demands to fulfill family responsibilities (Tseng, 2004), suggesting that even sources of resilience may exacerbate stress under certain circumstances.

Notably, the bulk of these studies were conducted with non-treatment seeking samples, and it is not well understood whether stressors experienced in community samples are the same stressors that prompt Asian American students to seek counseling services. Our search for studies that have examined the presenting problems of clinical populations of Asian American college students yielded only a few studies. Atkinson et al. (1995) found that Asian American undergraduate students were more likely to seek counseling for academic problems compared to personal problems. Kim et al. (2016) found that the most common presenting problems endorsed by Asian American college students initiating counseling services were academics, depression, anxiety, and interpersonal relationships. In addition, Wong et al. (2011) found that recent family, academic, and financial problems were the top three stressors endorsed by students who had seriously contemplated attempting suicide in the past 12 months. Stokes et al. (2021) found that concerns about ethnic/racial discrimination, adjustment to the university, irritability/anger/hostility, and test/speech/performance anxiety contributed to greater levels of distress among international Asian and Asian American students compared to European American students. However, these studies utilized self-report check-list measures which do not capture problems that may have been culturally or contextually distinctive for Asian Americans. Professional mental health providers may be able to provide analytic detail of the range of psychosocial factors that impact Asian American college student mental health through their clinical conceptualizations and insights gained over the course of treatment.

With regards to the course of treatment with Asian American students, some research suggests that even after engaging in college counseling services, Asian American students’ distress decreases the least over the course of treatment compared to their African American, European American and Latino counterparts (Lockard et al., 2013; Stokes et al., 2021). Others have found that while treatment outcomes are similar for Asian Americans compared to clients of other races/ethnicities, Asian American students tend not only to exhibit greater severity of distress at the start of treatment, but also continue to have clinically significant distress at termination (Kearney et al., 2005; Kim et al., 2016). These findings highlight the need for greater research on what treatment challenges are readily apparent and what approaches may be most effective for treatment seeking Asian American college students.

With regards to specific treatment approaches with Asian Americans, there is a common perception that Asian Americans prefer directive therapy and are less likely than European Americans to tolerate ambiguity in treatment (Sue & Sue, 2016; Wong et al., 2007). Conceptually, Asian cultural values, such as hierarchical relationships and respect for authority figures, may lead Asian American students to see therapists as expert authority figures who can help solve their problems (Hwang, 2006). Some research evidence also suggests that those who expect therapy to be directive tend to find directive therapists to be more credible than non-directive therapists (Wong et al., 2007). While these findings seem to suggest that Asian Americans may be more responsive to concrete problem-solving and directive therapies versus insight and process-oriented therapies, other research suggests that the relationship may be dependent on other factors such as the client’s cultural identification and self-construal (Wong et al., 2003), the therapist’s multicultural competence (Wang & Kim, 2010), and ethnic match between therapist and client (Ibaraki & Hall, 2014; Meyer et al., 2011). For example, one study found that Asian American college students who had low levels of identification with White identity rated Cognitive Therapy to be more credible than Time Limited Dynamic Therapy (Wong et al., 2003). Ibaraki and Hall (2014) found that ethnic match increased treatment stays and reduced attrition among racial minority college students, presumably due to enhanced perceptions of therapists’ shared worldview and multicultural competence, which has been found to be associated with greater client perceptions of therapist’s empathy and credibility, and stronger therapeutic relationships (Kim et al., 2005; Meyer et al., 2011; Wang & Kim, 2010). Taken together, these findings suggest that while the effectiveness of diverse treatment approaches may differ for Asian Americans, multiple cultural and contextual factors can also lead to possible intragroup differences in treatment preferences and engagement.

In this study, we explored therapist narratives of their work with Asian American clients in a college counseling center using qualitative data to expand on the body of existing research, most of which is quantitative. The first aim was to explore the unique presenting problems among clinical populations of Asian American college students from the perspective of the therapist. The second aim was to explore what treatment approaches and processes therapists have found most useful in treating Asian American college students. Due to the exploratory nature of these aims and the corresponding methods used for data collection and analyses, no specific predictions were made regarding the results.

## Method

### Participants

Our sample consisted of 17 licensed therapists at the counseling center of a large public university on the West Coast. This sample size is within the recommended range for studies employing a consensual qualitative research (CQR) framework (Hill et al., 1997). The racial/ethnic breakdown of the sample was 9 European Americans, 5 Asian Americans, and 3 who identified as belonging to other racial/ethnic minority groups. Data were not collected on ethnic subgroup identification within these discrete categories. There were 5 males and 12 females. Four therapists were licensed at the master’s level, and the rest were doctoral level providers. No additional demographic data were collected other than race/ethnicity, gender, and level of licensure.

### Procedure

Participants were recruited using purposive and convenience sampling at a university counseling center. Efforts were made to capture a range of therapist theoretical orientations, level of training, and experience working with Asian American students. Potential participants learned about the research study via a presentation by the principal investigator (last author) and were informed that the purpose of the study was to better understand the needs of Asian American college students at the counseling center to aid in program development. The voluntary nature of the study was emphasized, and clinicians were informed that their decision to participate would have no implications for their performance review or professional advancement. Interested individuals were invited to contact the research team for an interview. After obtaining informed consent from the participants, the principal investigator conducted hour-long semi-structured interviews in which participants were asked the following questions:

1. Given your experience working with Asian American clients at the counseling center, what are some of the distinctive concerns or problems they present? In your experience, are there problems or risk factors to which Asian American students are particularly vulnerable?
2. I’d like you to tell me about a particularly memorable or representative Asian American client with whom you’ve worked. I’d especially like to hear about a client for whom matters of Asian American cultural, familial, or context were notable. What brought that client in for counseling?
3. What treatment strategies or approaches have you found most effective in your work with Asian American clients?

Since the interview was semi-structured, the interviewer probed for further details about specific case experiences to elicit examples, but the main structure of each interview did not deviate from the three questions listed above. As such, the data generated from each interview was consistent in the general topics covered. All study procedures were approved by the university’s Institutional Review Board prior to recruitment and data collection. All interviews were digitally recorded and transcribed prior to analysis. Incentives were not provided for participation.

### Analytic framework

A CQR framework was adopted to inform the analytic approach (Hill & Knox, 2021), as the goals of this project were to elicit and inductively analyze the experiences and attitudes of participants in response to each open-ended interview question. The semi-structured interview format allowed participants to contribute to the flow of the discussion and reflect more deeply on their experiences. We also sought to understand *how* therapists approached their work with Asian-American clients, and CQR is well suited to these aims (Hill & Knox, 2021).

### Research team and training

The research team consisted of the primary investigator (fifth author), two coders (third and fourth authors), and two auditors who also took the lead on writing this manuscript (first and second authors). The first author identifies as a Taiwanese American cisgender female who immigrated to the United States in early childhood. She is a licensed psychologist with training in quantitative and qualitative research and worked for a few years in college counseling. The second author is a South Asian American cisgender female with a background in both quantitative and qualitative research, pre- and post-doctoral experience in college counseling, and is licensed as a psychologist. She was born and raised in the United States. The third author is a first-generation Chinese American who was raised in Hong Kong until young adulthood. She is a clinical psychologist with training and experience in qualitative and mixed methods research. The fourth author is a clinical psychologist of Chinese descent who immigrated to the United States in her late adolescence. Both the third and fourth authors completed a year-long clinical placement as graduate student therapists at the university counseling center. The fifth author identifies as a cis woman of Hakka Chinese descent, born in Jamaica and raised in Canada by immigrant parents. She is a clinical psychologist with postdoctoral training and mentored experience in qualitative and mixed methods health services research.

When the study was conducted, the coders were graduate students and had little prior experience with qualitative data analysis. The coders were trained by reviewing relevant literature and engaging in the process of the study in consultation with the primary investigator. Both auditors had prior experience conducting qualitative research, which informed their roles as auditors. All members of the research team had equal opportunities to provide input on the final manuscript.

### Coding of transcripts

All interviews were transcribed verbatim and uploaded to Dedoose (2022), a qualitative analysis software, in which all subsequent coding and analysis was conducted. Before coding began, each interview transcript was de-identified and divided into meaningful units for analysis which we termed excerpts. Two members of the research team (third and fourth authors) read all transcripts and generated a list of core ideas and possible domains over a period of several weeks. Meetings between the coders and principal investigator (fifth author) were held on a regular basis to discuss emerging observations and then to engage in the cross-analysis process which produced a preliminary set of domains and categories for coding the data, as per the recommendation of Hill and Knox, (2021). The principal investigator encouraged the expression of viewpoints during coding meetings in order to reduce the potential impact of power dynamics on the team (the coders were graduate students at the time). The coders also reviewed the data independently to develop initial codes before discussing their ideas and reaching consensus on the codes, thereby reducing the potential for groupthink and increasing the trustworthiness of the process.

Subsequently, the first and second authors joined the research team as internal auditors and reviewed the domains and categories that the other team members had developed. Neither the first nor the second author were involved in any prior procedures of the study and therefore brought an “outside” perspective. This arrangement served to introduce diverse viewpoints on the team and enhance trustworthiness of the method. In terms of process, both auditors had worked with all other members of the research team in varying capacities before this project, and this existing rapport allowed for the discussion of feedback within a respectful and trusting dynamic, which is critical to the iterative process of data analysis and auditing in CQR, since the team itself can be considered an instrument (Hill et al., 1997). The auditors reviewed the process by which codes were developed, and the coherence and stability of the findings. After this internal audit, the primary coding team discussed the feedback with the internal auditors with the goal of achieving consensus on a final coding list. Some of the adjustments after the internal audit included combining codes that frequently co-occurred, removing codes that were too narrow or infrequent to contribute meaningfully to our analysis, and making minor adjustments to domain and category names.

In coding, multiple domains could be applied to the same excerpt, and in some cases, one or more categories within the applied domain code was also assigned to an excerpt. If a category code was applied, then the corresponding domain was also coded, but a domain code could be applied without a category code if the content did not fit within a category. After the coding list was finalized, the application of the codes to the remaining transcripts was done independently and consistently by the two coders such that inter-rater agreement was 93.8% and Cohen’s kappa was .80 across all levels of codes.

## Results

The final list of codes comprised seven domains, each of which had a minimum of two to a maximum of seven categories. The final coding list, frequency of codes, and frequency label for each domain and category are presented in Table 1. The frequency label captures the representativeness of a code because it is determined by the number of participants who discussed the code’s content, regardless of the frequency with which it was discussed across participants (Hill et al., 1997). For our sample size of 17 therapists, the following frequency labels were used: General (16–17), Typical (9–15), Variant (4–8), and Rare (1–3). These labels are presented in parentheses in the sections below for all domain and category codes. Based on the number of codes that were in the General and Typical ranges, we determined that our data were overall representative to the sample (Hill & Knox, 2021).

**Table 1.** Code descriptions and frequencies (total number of domain Codes = 302)

Domain and Category Codes	Frequency (%)	Representativeness	Description
**Stereotypes and discrimination**	22 (7.28%)	Typical (9)	Feels subjected to and negatively impacted by stereotyped expectations and biases. Perceived ethnic/racial discrimination.
Gender and sexuality	17	Variant (8)	Influenced by body image expectations, feminine or masculine stereotypes.
Unfair treatment	1	Rare (1)	Perceives poor treatment, bias, or rejection by others due to their ethnicity.
Model Minority Stereotype (MMS)	2	Rare (2)	Pressure to conform to elevated academic performance expectations as an Asian American. Internalization of MMS, and maladaptive perfectionistic tendencies.
Prejudice or bias	2	Rare (2)	Indications of prejudice or biased feelings toward other groups.
Internalized inferiority beliefs	4	Rare (2)	Holds a negative self-concept related to their Asian background (e.g. low self-esteem related to ethnic identity).
**Family stress**	64 (21.19%)	General (16)	Stress originating with student’s immediate or extended family members.
Obligation	36	Typical (14)	Stress related to filial obligation, including pressure to succeed to meet the needs or expectations of family.
Estrangement	12	Variant (7)	Lack of a nurturing or meaningful relationship between parents and the student (e.g. alienation, lack of support).
Conflict	13	Variant (8)	Overt conflict typically between parents and the student with varying topics (e.g. parental authority vs. autonomy).
Family assistance	7	Variant (5)	Being burdened with familial responsibilities (e.g. problem solving, caregiving, providing instrumental help).
**Acculturative stress**	9(2.98%)	Variant (8)	Immigration-related stress, first-generation stressors.
Social isolation	5	Variant (4)	Social isolation and loss of social support network due to immigration.
Communication and language barriers	5	Variant (5)	Communication and language barriers in some areas of life.
**Emotion regulation**	41(13.58%)	Typical (13)	Problems with managing emotional experience.
Emotional expression	7	Variant (6)	Paucity or immaturity in expressing internal emotional states and communication.
Emotion identification	13	Typical (9)	Lack of insight and trouble identifying emotional experience.
Emotion socialization	13	Typical (9)	Differences in emotion perception and expression due to being reared in a culturally distinct environment. Family lacks support in emotional communication.
Extreme negative coping (acting out)	12	Variant (5)	Maladaptive ways of coping or responding to intense negative affect (e.g. self-injury, high risk sexual behavior, substance use, gambling, violent relationships).
Avoidance	7	Variant (4)	Extreme avoidance patterns and absence of goal-directed behavior to manage negative affect (e.g. social withdrawal, school avoidance, non-stop gaming).
**Academic stress**	38 (12.58%)	Typical (13)	Stress due to academic performance or pathways (subjective or objective failure).
Poor performance	8	Variant (5)	Objective indicators of poor performance (e.g. academic probation, risk of dismissal, displacement from a program).
Subjective distress about performance	7	Variant (4)	Student is not living up to own standards of achievement.
Dissonance about field of study	5	Variant (4)	Concerns or dissatisfaction about choice of major or current area of study, or confusion about academic direction.
**Identity search**	53 (17.54%)	General (16)	Struggle with achieving identity in relevant domains (e.g. search for meaning, conflict between choices without ability to integrate paths, lack of sense of self).
Developing bicultural competence	13	Typical (9)	Deficits in functioning as alternately independent (American) or interdependent (Asian) as the situation calls for, including home-school transition problems.
Professional/Academic identity	9	Variant (7)	Internal conflict a choice of major, lack of satisfaction or fulfillment, misfit with competence, or in response to intense family pressure for an area of study.
Interpersonal/Social belonging	27	Typical (10)	A sense of confusion about where the student belongs. Includes formation of peer or romantic relationships. Lacks feelings of acceptance socially.
**Treatment approach or considerations**	75 (24.83%)	General (17)	Characterizations of treatment techniques, orientations, elements, or processes.
Directive intervention	33	General (16)	Behavioral, cognitive behavioral, skills oriented.
Family communication	9	Variant (6)	Focus on building communication within the family.
Ethnic match favorable	13	Variant (8)	Discussions that include advantages of clinician-student match in race/ethnicity.
Psychoeducation	7	Variant (5)	Education about mental health and illness, intervention for stigma concerns, and as a part of intervention orientation.
Insight oriented intervention	32	Typical (13)	Humanistic, psychodynamic.
Ethnic match unfavorable	9	Variant (6)	Discussions that suggest that client-therapist match presents a disadvantage.
Group therapy	9	Variant (6)	Considerations about the fit of group therapy for Asian American students.

### Presenting concerns

Across all interviews, family stress (general) was the most frequently discussed presenting problem, followed by identity search (general), emotion regulation (typical), academic stress (typical), stereotypes and discrimination (typical), and acculturative stress (variant). Family stress was also one of the most representative domains, being discussed in all but one interview. Our coding of the interviews revealed that these presenting concerns were frequently discussed as interconnected and contextual, providing a more complex picture of how these presenting concerns are experienced and discussed in therapy. The domain of family stress included the category codes of obligation (typical), conflict (variant), estrangement (variant), and family assistance (variant). Several therapists described working with students whose desire to succeed academically and professionally was motivated by the goal of financially supporting their parents. The theme of Asian American students assisting their families with various tasks “at home” was also discussed frequently, particularly in cases where a student’s parents were not fluent in English. For example, one therapist described how a student’s family obligations were particularly burdensome given her mother’s gambling:

Therapist 11 (Other Ethnic Minority, Female): And then mother gambled away about $30,000 worth of the money and the expectation is now that this student take care of not only finishing school but now has to take care of handling the bankruptcy, handling the English … there is no real English-speaking parent.

Another therapist described working with an Asian American client who was struggling with procrastination and poor academic performance, concerns that were fully understood only when the client’s family context came to light:

Therapist 4 (Other Ethnic Minority, Female): I just started to explore more and more about what it would mean if he did graduate, what would that look like … (and we) talked too about … he had a lot of siblings; he lived in this little tiny house, all these siblings; no one was doing anything except him, pretty impoverished family, and they were all waiting for him to graduate. And so, it was just too much for him.

While these examples illustrated multiple negative aspects of family stress, some therapists also discussed how the context of family stress could simultaneously confer risk and protection to Asian American students:

Therapist 9 (European American, Female): I guess one thing I’ve heard several times is the feeling that there’s going to be a responsibility to parents … and I’ve heard that range from I want to have a good job so that I can later support my parents or even recently someone who was suicidal didn’t want to kill herself because she felt a responsibility to eventually provide for her parents.

Thus, even though family stress was the most frequently discussed presenting concern by the therapists in our study, the nature and impact of the family stress varied based on the client.

The second most common domain, identity search (general), was discussed in all but one interview and included concerns about interpersonal belonging (typical), bicultural competence (typical) and professional or academic identity (variant). Several therapists described Asian American students as struggling to be autonomous from their families in many ways, such as deciding upon their own academic and career goals despite strong family pressure to pursue specific fields. One therapist discussed some Asian American students leading “double lives” to fulfill their own desires and those of their families:

Therapist 15 (Asian American, Female): Some of them will resort to more of a double life or passive-aggressiveness … that’s how they find the middle ground, but they do whatever they please or they live out a life of their own choosing, but outwardly … or especially in front of their family, they present a totally different aspect.

The third emerging presenting problem was emotion regulation, which was typical in our sample in terms of representativeness, and often included emotion identification (typical) and emotion socialization (typical). One therapist postulated that some Asian American students seemed to be less emotionally expressive because they did not learn how to access or relate to their feelings in their families of origin. Another therapist noted how the difficulties of identifying, acknowledging, and expressing emotions are often intertwined with and complicated by family stress and identity search concerns:

Therapist 15 (Asian American, female): … the resentment is just barely under the surface and yet acknowledging that creates so much guilt and shame on their part … that they [the parents] sacrificed so much … who am I to just pursue a selfish life of my own choosing? Sometimes, they follow the parent’s prescription, which obviously did not bring them the anticipated success and happiness, and yet it’s very, very hard for them to openly acknowledge that they’re angry with their parents. They may choose a very self-destructive path …

Another presenting concern among Asian American students was the domain code of academic stress (typical) which was discussed in 13 of the 17 interviews. However, many therapists noted that while academic stress may at times be the presenting concern, the therapeutic process can sometimes reveal several layers beneath a student’s initial presenting concern to include family and relational problems and other personal struggles:

Therapist 2 (Asian American, female): After some exploration they realize that academic stress isn’t truly the heart of the problem because it’s some personal struggle that’s actually affected their academic performance … I guess sometimes you just don’t take the face value initially presented with, really needed to go further in-depth exploration to [understand] what’s going on.

This therapist’s example again illustrates how many of the presenting problems are significantly intertwined and complex and can take time to uncover.

Within the domain of stereotypes and discrimination (typical), which was discussed in nine interviews, gender and sexuality (variant) was the most frequently coded category, followed by internalized inferiority (rare), prejudice or bias (rare), model minority stereotype (rare), and unfair treatment (rare). The nature of the gender/sexuality concerns included pressure to “conform to a thin petite body size” for women, and feelings of inadequacy due to “small stature” for men. Therapists noted that even as these themes commonly surfaced in their work with Asian and Asian-American students, it wasn’t clear if the frequency at which these concerns arose was any different from other ethnic groups, or other students in general.

Therapist 7 (European American, female): One of the things I’ve seen some gay Asian American males who have struggled with identity issues and family pressures and expectations and really been conflicted about feeling with their families but also just in terms of their attractiveness towards other gay men that’s been a theme with them.

Acculturative stress (variant) was coded only nine times across all interviews but was discussed by eight participants, and the content was in the categories of social isolation (variant) and language barriers (variant). Acculturative stress was noted to not only contribute to social isolation, but also low self-confidence. An example is provided below (therapist is referring to an Asian American client in the first sentence):

Therapist 7 (European-American, female): They were in places where there weren’t many Asians … so that made it more alienating and feeling set apart and different and hard to find a support system. So usually what I do is go back even with someone with their self esteem and [ask] what it was like in school, in growing up, and how they fit in … how they were accepted. The other part of that is the language issue. If they were born here and if they grew up speaking English it was one thing, but if they came and had to learn the language that made a difference and what age that happened.

### Treatment considerations

All participants were asked about treatment considerations (general) when working with Asian American clients, so this domain was coded in all interviews, and approximately one quarter of the content across all transcripts pertained to treatment considerations. As Table 1 indicates, the frequency with which therapists discussed using directive (general) or insight-oriented approaches (typical) was nearly identical, suggesting there was no single most frequently used approach by therapists in our study when working with Asian and Asian American clients. Some therapists did describe more CBT-oriented, directive work. For example, one Asian American female therapist described how she uses cognitive behavioral interventions in helping her clients feel empowered:

Therapist 15 (Asian American, female): I could be very, very CBT. I really, really believe in environment, and you caught me saying the courage word quite a few times because, actually, that’s a central, intervention for me … empowerment, learning to take risks, and also learning to look at things from a fresh set of eyes instead of operating from some old belief system that’s just no longer functioning. I also oftentimes talk about freedom of choice, and I feel like a lot of times people underestimate the fact that they actually have far more freedom than they’re aware of and so they’ve been operating according to a very, very limited set of choices and belief system, and so they’ve been imprisoning themselves, and so once they realize that they have more options, and then they evaluate the pros and cons.

In contrast, some therapists described more insight-oriented approaches. An Asian American male therapist described how he implements an insight-oriented approach that includes a discussion of values to facilitate resolution:

Therapist 13 (Asian American, male): I don’t see my role as challenging their values but rather trying to understand them and at what point or what juncture does it become a conflict or problematic or what else are they trying to juggle? So I address it in that context of identifying what are their other values, and how do those conflict, like maybe they feel obligated to go home over the weekends but what else … well, I want to go over here and go to that party or whatever, so … just to address it in that way … but … I don’t see my role as guiding them or telling them what to (do).

While some described more insight-oriented work and others described more directive, CBT oriented work, it was clear that therapists often integrated varying approaches to produce a flexible approach to address each client’s individual needs. One Asian American female therapist provided a more detailed description of integrating insight-oriented approaches with cognitive and behavioral approaches:

Therapist 1 (Asian American, female): Talking about the feelings and giving them some space … and empathizing … I think that’s really important, particularly with people who are not used to talking about those feelings or don’t trust their feelings to just really validate what they’re experiencing … so I might start off really slow … and then I do a lot of emotion regulation work. I might use mindfulness if they can tolerate that say … (sitting) … and being mindful … and just when they’re feeling calm, so that they can learn this idea of sitting with, when they’re feeling things more intensely … maybe (think) about what are they afraid will happen if they do feel things intensely …

In addition to directive and insight-oriented approaches, therapists discussed integrating other treatment modalities such as psychoeducation (variant), family therapy (variant), and group therapy (variant) for their Asian American clients. One therapist described situations in which she might provide psychoeducation about the counseling process due to stigma:

Therapist 14 (European American, female): Sometimes, there’s a lot of anxiety around accessing the treatment to begin with. I have to kind of do some psychoeducation about counseling and treatment with them that … there still can be kind of a perception of treatment as only for people that are really disturbed, and some of them are reluctant to let family members know that they’re coming in for treatment.

Another therapist described a specific case in which she integrated family therapy, which resulted in a better outcome for her client:

Therapist 2 (Asian American, female): I really felt the usefulness of family therapy because that’s their community, that’s the major support of resources and understanding. It is complicated because I think Western culture emphasizes so much about individual efficacy. The Asian culture is still family oriented; if we could utilize it for the positive, it’s actually going to be very healing and powerful.

Overall, these examples illustrate how the therapists integrated a variety of approaches, informed by cultural considerations, to address the myriad of needs of Asian American students.

Notably, in addition to discussing these treatment approaches, most therapists also spoke about how ethnic match was either favorable (variant) or unfavorable (variant) in working with their Asian American clients. One Asian American female therapist spoke of experiencing her racial/ethnic identity as an advantage in working with Asian American students:

Therapist 15 (Asian American, female): And I think in a (way) … being a Chinese-American therapist actually I think it’s an advantage because I actually could talk about … individualized … choice … or decision-making without sounding like I’m culturally … insensitive.

However, another Asian American therapist noted that it wasn’t always clear whether or not ethnic match was favorable or unfavorable. She spoke of how she inquires about the client’s perception of the therapist’s racial/ethnic identity, even when the client is Asian American:

Therapist 3 (Asian American, female): I think it’s important to talk about the therapeutic match with the client. Even if I am Asian and if they are … Just the small acknowledgement, like, “Oh, these are some of the things that we can talk about”, or “you feel like I can understand you” … something like that. Sometimes you know it’s very apparent from the very beginning that they’re really awkward with me because I’m Asian … and so the first interview might sort of go … “how do you feel about working with me”, and I would add, “how do you feel about working with me especially because I’m Asian-American … ” to see how that feels and you know … go on from there.

Additionally, therapists who did not identify as Asian American described that they did not necessarily find themselves at a disadvantage in establishing rapport with their Asian American clients. For example, one European American female therapist spoke of her surprise when one international student she worked with stated that she preferred to work with an “American” therapist as opposed to an ethnically matched therapist. Another European American female therapist discussed how her own identity as an immigrant influenced her ability to understand immigrant students’ experiences, and how she takes care to develop rapport and trust:

Therapist 10 (European American, female): I’ve found that through self-disclosure that I’ve come through an immigrant background and understanding dynamics of family and communal aspect and … just sort of not even that the community dynamics are similar necessarily, but just having that understanding and awareness … that has really allowed me to sort of create relationships with some of the students. And then even just in terms of empathy, just sort of acknowledging that there is no rush for them to trust me. People [can] take the time that they take to feel trust, and I will continue to reassure them that this is private and confidential.

The diversity of experiences that therapists reported with regards to ethnic match and mismatch revealed how experiences varied according to the specific therapist-client dyad, suggesting that the influence of ethnic match on treatment outcome is likely moderated by numerous contextual and therapeutic dyad-specific variables.

#### Summary

Therapists reported a range of presenting concerns based on their work with Asian American students, and these concerns were often interrelated. One notable characteristic of the therapists’ narratives is that their clients who indicated the same category of concern (e.g. family obligation) may have had very different experiences of the concern itself, and in some cases, the “concern” was not necessarily problematic in all contexts. In keeping with the complexity of results for presenting concerns, therapists also reported using different therapeutic approaches according to the needs of the client, cultural considerations, and their own clinical judgment. Themes regarding ethnic match or mismatch also surfaced during the discussion of treatment strategies and approaches, with many therapists reporting explicitly discussing the match or mismatch with clients. However, it appears that whether an ethnic match is favorable or not depends on more than demographic similarity, at least from the perspective of therapists.

## Discussion

The qualitative nature of the methods used in this study allowed an in-depth analysis of therapists’ reports of the issues they commonly encountered when working with Asian American clients, as well as their process in selecting and implementing interventions. Our analyses revealed that the presenting concerns most frequently encountered when working with Asian American clients were multi-faceted, intertwined, and contextual, an insight only revealed by examining our qualitative data in context. In discussing treatment approaches, therapists reported integrating various approaches in their conceptualizations to best fit the needs of the client and addressing how ethnic match or mismatch may be favorable or unfavorable. These results suggest that the impact of treatment approaches and ethnic match on treatment process and outcome is contextual and case-specific and warrants further qualitative exploration.

Our finding that family stress frequently co-occurred with academic and identity issues is consistent with previous studies that have examined the types of presenting concerns that Asian American students report (Ibaraki & Hall, 2014; Lockard et al., 2013). The prominence of family stress as a presenting concern reported by the therapists in this study is also consistent with the finding that for Asian Americans, high levels of family conflict may be a precipitating factor for seeking formal mental health treatment or correlated with depression and anxiety (Tummala-Narra et al., 2021). In this study, some therapists reported that family obligation at times hindered their clients’ identity development and academic performance. On the other hand, some therapists also reported that family obligation could serve as a protective factor for students struggling with depression or adjustment concerns. These data suggest that while Asian American clients may often present with family and academic stressors, the way these stressors function may vary, and therefore, careful assessment by the therapist is warranted to avoid over-pathologizing a client’s relational dynamics, or under-pathologizing by assuming a familial dynamic is adaptive when it may not be.

Even though the interview questions did not ask specifically about ethnic match, nearly all therapists who discussed the topic reported that they addressed ethnic match explicitly with their Asian American clients, which led to useful clinical information. The therapists also reported that how a client experienced the match or mismatch varied on a case-by-case basis. Some therapists noted that they discuss ethnic match with all clients, not just those who are Asian American. For instance, one Asian American female therapist said,

Therapist 2 (Asian American, female): If it’s cross culture, I would say clinician needed to really check in with (them)…it’s like, with Asian-American too, it’s like how do you feel about working with me? I’m coming from a different cultural background. I’m wondering what are your thoughts, about any concerns you have? I think that can really open up dialogue … I check in not just with Asian-American students like … you can’t believe how often actually when I ask African-American students I work with, and I, as you can see … I am an Asian-American woman, how would you feel working with me? What difference would that make if I’m from a different cultural background?

Similarly, one European American female therapist described her approach in addressing cultural differences with her clients:

Therapist 14 (European American, female): Well, I know that it does for me … as a clinician because (if) there’s a cultural difference, then … I really … want to be very mindful of my understanding of that person and where they are in their cultural awareness, so I will … proceed I think more slowly just so that I don’t overstep, or I don’t misunderstand, and I really want them to feel comfortable with me and see if they do or not and sometimes that takes a while.

These quotes are representative of other therapists’ discussions of ethnic match, suggesting this is an important clinical issue to assess and directly discuss with clients. As other scholars have suggested, ethnic match may be a proxy for cultural match or shared experiences (Ibaraki & Hall, 2014; O. Meyer et al., 2011), and exploration by the therapist and client can help determine what aspects of this match may be helpful to the client. Community studies have found that bilingual providers who are fluent in both the language and cultural nuances of the particular ethnic or cultural group can aid in participation in treatment, although some studies have found that ethnic match is no longer a significant clinical predictor of decreasing dropout after the first session once rapport is established (Maramba & Nagayama Hall, 2002). Similarly, some meta-analytic reviews on racial/ethnic match have found that although clients tend to prefer therapists of their own race/ethnicity and view them more favorably than other therapists, there are no significant benefits to treatment outcomes (Cabral & Smith, 2011; Shin et al., 2005). And yet, a more recent meta-analytic study found that Asian Americans are much more likely to increase participation in mental health treatment than other racial groups when racially matched with a therapist (Smith & Trimble, 2016). Given the inconclusive results in the empirical literature and the findings in the current study, we suggest that no straightforward assumptions can be made regarding the therapeutic outcomes of ethnic match, which may vary by individual client and client-therapist dyads.

Our findings on treatment approaches revealed that the therapists in our study discussed using directive approaches and insight-oriented approaches with the same frequency, which portrays a different picture than the common perception that Asian American clients desire and are more responsive to directive approaches (D. W. Sue & Sue, 2016). In fact, one therapist described this perception as a *“stigma”* that may prevent therapists from employing insight-oriented approaches with their Asian American clients, even when the client could benefit from such approaches. Therapists also described the integration of adjunctive forms of therapy, such as group therapy and family therapy, to support their individual work with Asian American clients. Little research has been done on the contributions of such adjunctive therapies in the context of college counseling, but the data presented here suggest that this may be a useful topic for further research on how to optimize the provision of services to this population.

### Limitations

Our findings are limited to the information gained from the therapist’s perspective. Future studies that examine the correspondence or divergence between client and therapist perspectives on main presenting problems and/or treatment would further illuminate the interactions between therapist and client, particularly by using qualitative methods. Another limitation is the design of this study as cross-sectional. We have no data on the outcome of the therapy and therefore are unable to systematically examine and conclude which approaches are most effective or lead to the most improvement in terms of treatment outcomes. Furthermore, although participants were asked what treatment strategies or approaches they found most effective in working with Asian American clients, they were not asked specific questions about cultural competence, cultural humility, or culturally adapted treatments and interventions. Hence, there may have been a lack of reporting on these aspects beyond more general discussion of treatment approaches and ethnic match by the participants.

Additionally, limited demographic information was collected from the participants. Future studies may consider collecting information about therapist’s ethnicity, theoretical orientation, background and training, and what percentage of their caseload consisted of working with Asian Americans, as this information may help further contextualize the study findings. The potential for participant self-selection bias may also limit generalizability. It is possible that therapists who were particularly invested in working with Asian Americans and more prone to use culturally adapted treatments or practices may have self-selected to participate in this study.

Another limitation of this research is that we did not explicitly seek to differentiate therapist experiences according to whether or not the client was an international student, or according to subgroups within the larger “Asian” racial group, which is highly heterogeneous. There is evidence that presenting concerns may vary systematically for International Asian students vs. Asian American students, and there may be unique adaptations that therapists use in working with International vs. domestic students (Stokes et al., 2021). Other researchers in this area have called for further attention to differences in psychological distress and the dynamics impacting treatment utilization among specific Asian subgroups (Park et al., 2020; Tummala-Narra et al., 2021) and we support this recommendation. Future research could also explore how perceptions of ethnic match may differ depending on ethnic subgroup identification, immigration status, and other cultural identities, and to what degree these perceptions impact treatment. Finally, although processes were in place to reduce biases in both structure and process, the backgrounds and experiences of research team members may have created potential coder biases. Potential biases, assumptions, and expectations were not formally documented during the process of coding, which is a limitation of this study.

### Clinical implications

The findings of this study highlight multiple implications for clinical work with Asian American college students. First, this study suggests that therapists should be attuned to the unique concerns that their Asian American clients may face and explore the intersections between various stressors and cultural factors in their clients’ lives. Our findings indicate that family stress is frequently reported by Asian American college students, and that it is often intertwined with academic stress. Therefore, while Asian American college students may frequently present with academic concerns, it is important to understand these concerns within the context of family stress. Therapists may prompt more specifically for issues such as family obligation, family conflict, and family estrangement, as these may be specific ways in which family stress is presented. Several therapists in our study also talked about exploring Asian American students’ values related to communication and conflict in the context of their families in order to develop culturally sensitive strategies for change. Furthermore, therapists should also consider asking Asian American college students about issues regarding identity search, emotion regulation, stereotypes and discrimination, and acculturative stress. These issues may sometimes be overlooked or not recognized by the client as clinically relevant. Some literature suggests that Asian American college students experience discrimination despite positive stereotypes (e.g. model minority stereotype) that may be ascribed to this group (Huynh & Fuligni, 2012). Therapists are also encouraged to examine any preconceptions of what Asian American college students’ presenting concerns typically are and to avoid making assumptions about how a student’s specific cultural and social context may impact their distress. For instance, one therapist in our study observed that acculturation to dominant culture norms is not necessarily correlated with a student’s nationality.

Second, addressing the cultural and racial/ethnic background of the client and what it means for them to be working with a therapist of a similar or different background directly in session appears to be helpful. Based on our findings, therapists are encouraged to have an open discussion with their clients about ethnic match/mismatch rather than to presume that ethnic match is favorable and mismatch unfavorable. This recommendation is especially pertinent given the heterogeneity of persons who may identify as Asian American. The racial category of Asian Americans comprises numerous ethnic subgroups from a vast array of countries and nationalities. For example, a client who identifies as Asian Indian may not perceive a racial/ethnic match with a Chinese therapist, even though they may both identify as Asian American. Similarly, cultural identity, immigration status, and acculturation can lead to intragroup differences in values, attitudes, and beliefs. Our suggestion to address racial/ethnic background and perceptions of match/mismatch in therapy is also consistent with research that has found the acknowledgment of issues related to race and ethnicity is particularly important to ethnic minority clients compared to White clients (O. L. Meyer & Zane, 2013). Likewise, previous research on Asian American students seeking college counseling suggests that therapists would do well to assess presenting concerns from the client’s perspective without making assumptions to formulate a comprehensive and culturally relevant conceptualization of the presenting concerns (B. S. K. Kim et al., 2009).

Lastly, our findings highlight that there seems to be no “one-size-fits-all therapy” when it comes to working with Asian American college students. While it was previously believed that directive/behavioral approaches were preferred by Asian American clients over more insight-oriented approaches, our findings suggest that it is rather dependent upon the needs and preferences of the therapist and client in their work together. According to the therapists in this study, both insight-oriented work and more directive/behavioral approaches can be beneficial, and in fact many described an integrative approach to counseling their Asian American clients. In addition, this study found that family and group therapy may also be beneficial approaches to working with Asian American college students and serve as appropriate supplements to individual therapy, depending on the needs of the student. As such, therapists are encouraged to consider and explore the diverse needs of their Asian American clients and to remain open to taking multiple treatment approaches.

## Data Availability

The data that support the findings of this study are available from the corresponding author upon reasonable request.
